# Dementia and psychotropic medications are associated with significantly higher mortality in geriatric patients hospitalized with COVID-19: data from the StockholmGeroCovid project

**DOI:** 10.1186/s13195-022-01154-w

**Published:** 2023-01-06

**Authors:** Juraj Secnik, Maria Eriksdotter, Hong Xu, Martin Annetorp, on behalf of the SweGeroCOVID Project, Aleksander Rytarowski, Kristina Johnell, Sara Hägg, Dorota Religa

**Affiliations:** 1 Center for Alzheimer Research, Division of Clinical Geriatrics, Department of Neurobiology, Care Sciences and Society, Karolinska Institutet, Neo, Blickagången 16, 14152 Huddinge, Sweden; 2 Department of Neurology, Charles University, Second Faculty of Medicine, Motol University Hospital, Prague, Czech Republic; 3 Theme Aging, Karolinska University Hospital, Stockholm, Sweden; 4 Department of Medical Epidemiology and Biostatistics, Karolinska Institutet, Stockholm, Sweden

**Keywords:** COVID-19, Dementia, Antipsychotics, Mortality

## Abstract

**Background:**

Dementia and psychotropic medications are discussed as risk factors for severe/lethal outcome of the coronavirus disease 2019 (COVID-19). We aimed to explore the associations between the presence of dementia and medication use with mortality in the hospitalized and discharged patients who suffered from COVID-19.

**Methods:**

We conducted an open-cohort observational study based on electronic patient records from nine geriatric care clinics in the larger Stockholm area, Sweden, between February 28, 2020, and November 22, 2021. In total, we identified 5122 hospitalized patients diagnosed with COVID-19, out of which 762 (14.9%) patients had concurrent dementia and 4360 (85.1%) were dementia-free. Patients’ age, sex, baseline oxygen saturation, comorbidities, and medication prescription (cardiovascular and psychotropic medication) were registered at admission. The hazard ratios (HRs) with 95% confidence intervals (CIs) of in-hospital, 30-day, 90-day, 365-day post-discharge, and overall mortality during the follow-up were obtained. Then, the associations of dementia and medication use with mortality were determined using proportional hazards regression with time since entry as a time scale.

**Results:**

After adjustment, dementia was independently associated with 68% higher in-hospital mortality among COVID-19 patients compared to patients who were dementia-free at admission [HRs (95% CI) 1.68 (1.37–2.06)]. The increase was consistent post-discharge, and the overall mortality of dementia patients was increased by 59% [1.59 (1.40–1.81)]. In addition, the prescription of antipsychotic medication at hospital admission was associated with a 70% higher total mortality risk [1.70 (1.47–1.97)].

**Conclusions:**

The clinical co-occurence of dementia and COVID-19 increases the short- and long-term risk of death, and the antipsychotics seem to further the risk increase. Our results may help identify high-risk patients in need of more specialized care when infected with COVID-19.

**Supplementary Information:**

The online version contains supplementary material available at 10.1186/s13195-022-01154-w.

## Introduction

The pandemic caused by the SARS-CoV-2 virus has led to more than 641 million cases and 6.6 million deaths as of December 7, 2022, with the omicron variant and its subvariant BA.5 dominant worldwide [1, 2]. Fortunately, the current trend in confirmed cases as well as deaths continues to decrease across the whole world [1]. In the Nordic countries, Sweden has taken a different approach to containing the spread of COVID-19, especially in the earliest stages, advocating mitigation of spread and recommendations, not direct lockdown measures [3]. The approach was scrutinized due to higher mortality in the first and second waves compared to the neighboring Nordic countries; however, it is not clear whether this was due to differences in the measures themselves [4].

Importantly, multiple contributors for severe COVID-19 outcomes have been identified (e.g., diabetes, hypertension, cardiovascular, and oncological burden) [5]. Dementia is an established predictor for higher mortality worldwide [6], and the patients may constitute a particularly high-risk subpopulation for both contracting the infection [7, 8] and complications of COVID-19 [9, 10]. This is likely due to a higher comorbidity burden [11], a higher susceptibility towards pneumonia [12], and the inability to comply with safeguarding measures, such as social distancing [8]. However, the associations between dementia and mortality risk in COVID-19 patients have been less consistent in the initial stages of the pandemic [5, 13]. Furthermore, the mortality studies suffer from significant heterogeneity in both statistical methods as well as the studied survival intervals. With a long-term follow-up of dementia patients who survived COVID-19 still scarce, and the expected worsening of cognitive decline not yet quantified, there is a further need for high-quality longitudinal studies.

Moreover, due to lockdown procedures and social isolation, the increases in neuropsychiatric symptom burden [14] and caregiver strain [15] likely contribute to higher psychotropic medication use during the pandemic [16]. The role of these medications, specifically antipsychotics in the COVID-19 setting, is controversial with both beneficial [17, 18] and negative findings reported [19, 20]. While the research on psychotropic treatments in dementia patients is still infrequent, emerging evidence shows increases in the overall use of antipsychotic medications [16, 21] and higher mortality risks [22].

Long-term monitoring of the survival and cognitive functioning in dementia patients will be necessary to gauge the overall impact of the COVID-19 pandemic.

Our group has previously shown that frailty may drive the higher risk of in-hospital mortality [23], which may have confounded the associations with dementia in previous research. In the current study, we aimed to investigate whether dementia diagnosis and psychotropic treatment (particularly antipsychotics) at hospital admission are independently associated with increased short- and long-term mortality among COVID-19 patients in Sweden.

## Material and methods

### Study design and study population

This was an open-cohort study based on patient data from the TakeCare electronic patient record system. The study base comprised 34,111 patients hospitalized in one of the nine geriatric clinics in Stockholm, Sweden (Theme Aging Karolinska University Hospital, Nacka sjukhus, Dalengeriatriken, Stockholms sjukhem, Löwetgeriartiken, Sollentunageriatriken, Sabbatsbergsgeriatriken, Handens sjukhus, and Jakobsbergsgeriatriken) during the period of October 18, 2019, to November 26, 2021. The earliest date of admission was left-truncated from February 28, 2020, and only patients hospitalized with COVID-19 diagnosis were included (as main or contributory diagnosis), and only the first hospitalization was considered. The final sample comprised 5122 patients, where 762 (14.9%) patients had dementia diagnosis at entry and 4360 (85.1%) were dementia-free (see Supplementary Fig. 1).

### COVID-19 diagnosis

Patients were assigned COVID-19 diagnosis if the patient records contained the ICD-10 code for confirmed (U07.1) or suspected (U07.2) COVID-19 infection. The presence of the SARS-CoV-2 virus was confirmed by the reverse transcriptase-polymerase chain reaction from the nasopharyngeal swabs as explained in detail in a previous article from our group [23].

### Dementia diagnosis

Dementia was assigned, if the electronic patient records contained either the ICD-10 codes F00-F04, F051, G30, G31, and A081 or the patient had been dispensed anti-dementia medication (ATC codes N06DA, N06DX01) at the time or prior to the first admission to the hospital.

### Medication

Prescriptions of beta blockers (ATC code C07), calcium-channel blockers (C08), renin-angiotensin-aldosterone system inhibitors (C09), statins (C10AA), antithrombotic medication (B01), antipsychotics (N05A), anxiolytics (N05B), hypnotics/sedatives (N05C), and antidepressants (N06A) were considered at admission or admission + 1 day.

### Confounders

Patients’ age, sex, comorbidities, and oxygen saturation in % (SpO_2_%) assessed at admission were extracted from the patient records. Attained comorbidities defined by the ICD-10 codes in the patient records were summarized in the form of the Charlson Comorbidity Index (CCI) [24], using the algorithm specified by Quan et al. [25].

The first, second, and third waves of COVID-19 were arbitrarily divided by the admission dates prior to and including August 31, 2020 (first wave); between September 1, 2020, and February 28, 2021 (second wave); and after February 28, 2021 (third wave).

### Mortality

In-hospital, post-discharge mortality (divided into 30-day, 90-day, 365-day, and overall post-discharge strata) and the total mortality that occurred during the follow-up were obtained from the patient data. When COVID-19 diagnosis was established, we considered all deaths to be due to COVID-19 infection. The follow-up for each patient started on the day of admission and was counted until either death or November 26, 2021—the end of the study follow-up. In the post-discharge mortality data, the analyses were performed on survivors of hospitalization.

### Statistical analysis

Age, points of CCI, and duration of hospitalization were analyzed as scale variables (were not categorized). All the other variables in the models were analyzed as binary/multicategory variables. For baseline comparisons, chi-square (categorical variables), independent-samples *t*-test (scale variables normally distributed), and Mann-Whitney *U*-test (non-parametric equivalent of *t*-test) were used to determine significant differences between the cohorts. The time from admission of the first hospitalization with COVID-19 diagnosis to death or end of study follow-up was used to determine total survival. The time-stratified analyses in survivors were censored on the discharge, 30-day, 180-day, and 365-day post-discharge as appropriate.

Survival was assessed as follows. First, we compared the crude mortality rates and mortality rate ratios between the COVID-19-positive patients with and without dementia in the overall follow-up as well as divided by COVID-19 wave strata. Second, Cox proportional hazards regression was used to determine the hazard ratios of dementia diagnosis and antipsychotic use on mortality, with increasing level of adjustment. In the unadjusted model, the variables were entered into the equation separately; model 1 was adjusted for dementia, antipsychotics, age, sex, and CCI categories, while model 2 was further adjusted for SpO2 at admission and prescription of statins, hypnotics, and sedatives at admission or day after admission. Proportionality of hazards assumption was assessed by introducing interaction-with-time terms in the models for each variable. If the assumption was not fulfilled, the results were presented as baseline and interaction effect for the variable. Time scale was time since entry in all models.

Furthermore, we performed multivariate Cox models of the association between dementia and total mortality in the strata of age groups, sex, and COVID-19 wave and between antipsychotic usage and mortality among dementia and dementia-free patients to test potential effect modification.

Data were analyzed using Stata v16 (Stata Statistical Software: Release 16. StataCorp LLC, College Station, TX) and R version 4.0.0 [26]. The threshold for significance was set at *α* level <0.05.

## Results

### Baseline characteristics

COVID-19 was the main cause of hospitalization in both dementia and dementia-free (82.7 vs 86.1%, *p* for overall difference <0.001), with other causes less frequent (Table 1). At admission, the patients with COVID-19 and dementia were older than dementia-free patients (84.3 vs 82.1 years). Moreover, the COVID-dementia group presented more frequently with oxygen saturation lower than 90% (8.6% vs 5.8%), while the comorbidity burden was distributed in the higher categories (21.0% vs 29.8% with CCI 2–3 points) in the dementia-free group. Moreover, the use of antihypertensive therapy was less frequent in patients with COVID-19 and dementia, while the use of antipsychotic (26.1% vs 8.3%), antidepressant (37.8% vs 23.9%), anxiolytic (39.9% vs 25.2%), and hypnotic/sedative (55.8% vs 47.5%) therapy was conversely more prevalent in COVID-19 and dementia patients.

**Table 1:** Baseline characteristics of patients with COVID-19 by dementia stat us

		COVID-19 yes (first admission with COVID-19)		
		Dementia (762)	Dementia-free (4360)	*p*
Age at admission, years		84.3 (7.4)	82.1 (8.6)	<0.001
Age group	<70	24 (3.1%)	335 (3.1%)	<0.001
	70–79	167 (21.9%)	1354 (31.1%)	
	80–89	362 (47.5%)	1729 (39.7%)	
	>89	209 (27.4%)	942 (21.6%)	
Male sex		345 (45.3%)	2080 (47.7%)	0.22
CCI group	0–1 points	572 (75.1%)	2576 (59.1%)	<0.001
	2–3 points	160 (21.0%)	1298 (29.8%)	
	>3 points	30 (3.9%)	486 (11.1%)	
SpO_2_	≥90%	688 (90.3%)	4050 (92.9%)	0.004
	<90%	65 (8.6%)	252 (5.8%)	
	*Missing*	9 (1.1%)	58 (1.3%)	
Beta blockers		311 (40.8%)	2283 (52.4%)	<0.001
Ca2+ channel blocker		192 (25.2%)	1354 (31.1%)	0.001
RAAS inhibitors		280 (36.7%)	2259 (51.8%)	<0.001
Statins		266 (34.9%)	1881 (43.1%)	<0.001
Antithrombotics		729 (95.7%)	4210 (96.6%)	0.22
Antipsychotics		199 (26.1%)	363 (8.3%)	<0.001
Antidepressants		288 (37.8%)	1043 (23.9%)	<0.001
Anxiolytics		304 (39.9%)	1100 (25.2%)	<0.001
Hypnotics/sedatives		425 (55.8%)	2073 (47.5%)	<0.001
COVID-19 wave	First wave	313 (41.1%)	1555 (35.7%)	<0.001
	Second wave	328 (43.0%)	1768 (40.6%)	
	Third wave	121 (15.9%)	1037 (23.8%)	
Main cause of hospitalization	COVID-19	630 (82.7%)	3756 (86.1%)	<0.001
	Cardiovascular	11 (1.4%)	123 (2.8%)	
	Respiratory	18 (2.4%)	113 (2.6%)	
	Neurological	58 (7.6%)	61 (1.4%)	
	External (trauma, poison)	20 (2.6%)	80 (1.8%)	
	Others	25 (3.3%)	227 (5.2%)	
Hospitalization duration, days		10.8 (6.8)	10.0 (6.9)	0.023
Discharged to home		316 (41.5%)	2904 (66.6%)	<0.001
In-hospital mortality		154 (20.2%)	394 (9.0%)	<0.001
Post-discharge mortality	30-day	73 (9.6%)	340 (7.8%)	0.097
	90-day	108 (14.2%)	466 (10.7%)	0.006
	365-day	166 (21.8%)	721 (16.5%)	0.001
	Overall	183 (24.0%)	796 (18.3%)	<0.001
Total mortality (in-hospital + post-discharge)		337 (44.2%)	1190 (27.3%)	<0.001

Age at admission is described as mean (SD). Other variables are described as *n* (%); SpO2 refers to the first oxygen saturation recorded during hospitalization. Attained comorbidities were recorded at admission and medication use was recorded at admission or at admission + 1 day. COVID-19 waves were divided based on dates—August 31, 2020 (first and second wave) and February 28, 2021 (second and third wave). Comparisons for scale variables across the dementia strata were performed using the independent-samples *t*-test (age at admission) and Mann-Whitney *U*-test (hospitalization duration). The chi-square test was used in all other descriptive analyses

*CCI* Charlson Comorbidity Index, *RAAS* renin-angiotensin-aldosterone system

### Mortality and the association with dementia and antipsychotic medication

**Fig. 1:**
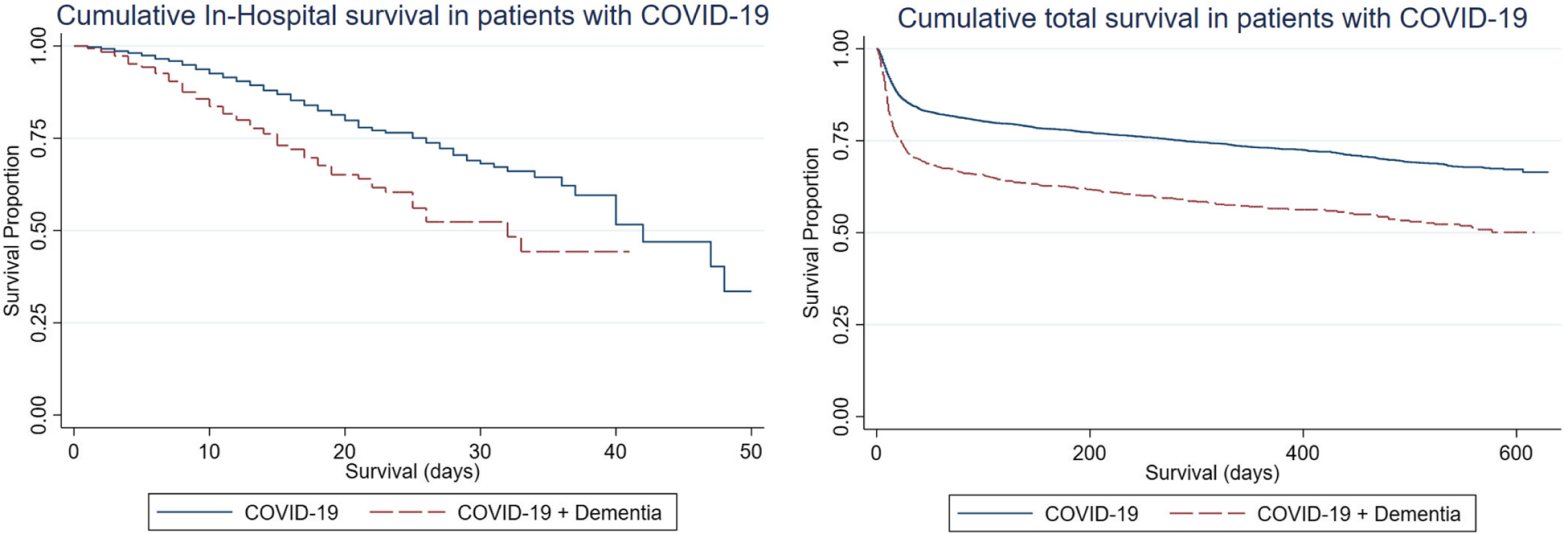
Kaplan-Meier cumulative survival curves during hospitalization and total follow-up by dementia status. Survival refers to overall survival during the whole follow-up

The baseline differences between the deceased versus survivors are summarised in Supplementary Table 1. Mortality rates in the dementia group were significantly higher across all time frames, with the overall mortality rate ratio of 1.88 compared to dementia-free patients with COVID-19. The largest difference in mortality was observed in the second COVID-19 wave, where the risk ratio was 2.39 (Fig. 1 and Supplementary Table 2).

In the multivariate analyses, dementia was associated with a 59% higher risk of total mortality during the follow-up [1.59 HR (95% CI 1.40–1.81)] when adjusting for all confounders (Table 2). Within the specific time strata, dementia was associated with 69% higher in-hospital mortality, 40% higher 30-day, 50% higher 90-day, 49% higher 365-day mortality, and 47% higher overall post-discharge mortality compared to dementia-free patients (Table 3). Furthermore, the use of antipsychotic medication at admission to hospital was associated with a 70% increase in total mortality [1.70 (1.47–1.97)]. Within the time strata, the antipsychotic use was associated with 3.58× higher in-hospital mortality at the day of admission [3.58 (2.43–5.26)] with the aggregate risk decrease of 5% per day of hospitalization [interaction with time 0.95 (0.91–0.98)]. In addition, antipsychotics were also associated with 49% higher 30-day, 48% higher 90-day, 46% higher 365-day, and 39% higher overall post-discharge mortality (Table 3).

**Table 2:** Risk of death during the whole follow-up in relation to dementia in patients hospitalized with COVID-19

			Patients with COVID-19 (*n*=5122)—hazard ratios (95% CI)		
			Unadjusted model	Model 1	Model 2
Dementia			1.83 (1.62–2.06)**	1.67 (1.47–1.90)**	1.59 (1.40–1.81)**
Age group	<70		0.73 (0.54–0.99)*	0.74 (0.55–1.01)	0.75 (0.56–1.02)
	70–79		Reference	Reference	Reference
	80–89		1.84 (1.61–2.12)**	1.83 (1.59–2.10)**	1.83 (1.58–2.10)**
	>89		2.81 (2.43–3.24)**	3.02 (2.61–3.50)**	2.90 (2.49–3.37)**
Male sex	Baseline effect		1.33 (1.18–1.51)**	1.35 (1.21–1.49)**	1.55 (1.37–1.76)**
	Male × time^a^		0.99 (0.99–0.99)*		0.99 (0.99–0.99)*
CCI group	0–1		Reference	Reference	Reference
	2–3		1.47 (1.31–1.64)**	1.50 (1.34–1.68)**	1.52 (1.35–1.71)**
	>3		2.31 (2.00–2.66)**	2.50 (2.17–2.90)**	2.56 (2.21–2.97)**
SpO_2_	Baseline effect	≥90%	Reference		Reference
		<90%	3.14 (2.60–3.81)**		3.00 (2.47–3.63)**
	SpO2 × time^a^		0.99 (0.99–0.99)**		0.99 (0.99–0.99)**
Beta blockers			1.28 (1.15–1.41)**		
Ca2+ channel blocker			0.81 (0.73–0.91)**		
RAAS inhibitors			0.86 (0.78–0.95)*		
Statins			0.71 (0.63–0.78)**		0.73 (0.65–0.81)**
Antithrombotics			1.13 (0.86–1.48)		
Antipsychotics	Baseline effect		2.14 (1.82–2.51)**	1.92 (1.67–2.20)**	1.70 (1.47–1.97)**
	Antipsychotics × time^a^		0.99 (0.99–0.99)**		
Antidepressants			1.16 (1.04–1.29)*		
Anxiolytics			1.37 (1.23–1.52)**		1.18 (1.05–1.32)*
Hypnotics/sedatives			1.35 (1.22–1.49)**		1.15 (1.04–1.28)*

^a^Sex, SpO_2_, and antipsychotic use violated the proportionality of hazards assumption in some models—presented as interaction mode where appropriate. Interaction with time represents the incremental change in the probability of death with advancing days of survival from the baseline probability; *p-value <0.05; **p-value <0.001. In the unadjusted model, the variables were entered into the equation separately; model 1 was adjusted for dementia, antipsychotics, age, sex, and CCI categories, while model 2 was further adjusted for SpO2 at admission and prescription of statins, hypnotics, and sedatives at hospital admission or day after admission. Results are based on the Cox regression models

**Table 3:** Risk of in-hospital, 30-day, 90-day, 365-day, and total post-discharge mortality in relation to dementia and antipsychotic usage in patients with COVID-19

		Patients with COVID-19—hazard ratios (95% CI)		
		Unadjusted model	Model 1	Model 2
In-hospital mortality				
Dementia		2.11 (1.75–2.54)**	1.81 (1.48–2.20)**	1.69 (1.38–2.07)**
Antipsychotics	Baseline effect	5.25 (3.67–7.51)**	4.67 (3.25–6.72)**	3.58 (2.43–5.26)**
	Antipsychotics × time^a^	0.92 (0.89–0.96)**	0.93 (0.90–0.97)**	0.95 (0.91–0.98)*
30-day post-discharge mortality				
Dementia		1.61 (1.25–2.08)**	1.46 (1.12–1.91)*	1.40 (1.07–1.83)*
Antipsychotics		1.54 (1.15–2.06)*	1.60 (1.18–2.16)*	1.49 (1.09–2.04)*
90-day post-discharge mortality				
Dementia		1.71 (1.39–2.11)**	1.58 (1.27–1.97)**	1.50 (1.20–1.87)**
Antipsychotics		1.59 (1.25–2.03)**	1.63 (1.26–2.10)**	1.48 (1.13–1.92)*
365-day post-discharge mortality				
Dementia		1.62 (1.37–1.92)**	1.56 (1.30–1.86)**	1.49 (1.25–1.78)**
Antipsychotics		1.56 (1.28–1.90)**	1.62 (1.33–1.99)**	1.46 (1.19–1.80)**
Overall post-discharge mortality				
Dementia		1.54 (1.31–1.81)**	1.52 (1.28–1.80)**	1.47 (1.25–1.74)**
Antipsychotics		1.46 (1.21–1.76)**	1.52 (1.26–1.84)**	1.39 (1.14–1.70)*

^a^Sex, SpO_2_, and antipsychotic use violated the proportionality of hazards assumption in some models—presented as interaction mode where appropriate. *p-value <0.05; **p-value <0.001. In the unadjusted model, the variables were entered into the equation separately; model 1 was adjusted for dementia, antipsychotics, age, sex, and CCI categories, while model 2 was further adjusted for SpO2 at admission and prescription of statins, hypnotics, and sedatives at hospital admission or day after admission. Results are based on the Cox regression models

### Subgroup analyses

In addition, the mortality risk associated with dementia was different across age and sex strata—the interaction terms of dementia with age and sex were statistically significant. Younger patients with dementia and male patients with dementia suffered from significantly higher mortality rates compared to their older and female counterparts, respectively (Table 4). Finally, the antipsychotic drug use was associated with similar mortality risks among the strata of dementia and dementia-free patients (Table 4).

**Table 4:** Overall mortality risk associated with dementia across the age, sex, and COVID-19 wave and antipsychotics in dementia

Patients with COVID-19—hazard ratios (95% CI)			
Strata: age, sex, wave		**Dementia vs dementia-free**	Interaction: dementia vs. age, sex, wave, antipsychotics
		Fully adjusted	
Age groups	Age ≤70	4.47 (1.96–10.2)**	0.82 (0.70–0.97)*
	Age 71–80	1.76 (1.28–2.43)*	
	Age 81–90	1.77 (1.47–2.14)**	
	Age >90	1.21 (0.97–1.51)	
Sex	Women	1.49 (1.23–1.79)**	1.29 (1.01–1.64)*
	Men	1.76 (1.47–2.11)**	
COVID-19 wave	First	1.37 (1.13–1.65)*	1.09 (0.91–1.31)
	Second	1.96 (1.61–2.40)**	
	Third	1.30 (0.86–1.96)	
Strata: dementia		**Antipsychotics users vs non-users**	
Dementia	No	1.80 (1.50–2.16)**	0.91 (0.68–1.22)
	Yes	1.42 (1.11–1.84)*	

The fully adjusted model included age at admission (in sex analyses); sex (in age analyses); comorbidity; oxygen saturation; use of statins; antipsychotic, anxiolytic, and hypnotic/sedative medication; and dementia. The interactions of dementia status with COVID-19 waves and antipsychotic usage were not considered statistically significant on the *α* level of 0.05; *p-value<0.05; **p-value<0.001

## Discussion

In this large cohort of geriatric patients hospitalized with COVID-19 infection in Stockholm, Sweden, the presence of dementia was associated with significantly higher in-hospital, short-term and long-term mortality compared to patients without dementia. Moreover, the prescription of psychotropic medication—particularly antipsychotics—was correlated with significantly higher mortality.

Dementia comprises multiple layers of pathology, clinical presentations, and prognoses; therefore, the outcome of COVID-19 infection may significantly vary among individual patients in the dementia spectrum, and the information on specific dementia diagnoses is currently insufficient. Importantly, multiple studies concur with the increased risk of mortality among dementia patients. However, the magnitude varies substantially—while the early report from Zheng et al. did not recognize dementia as a major prognostic factor [5], 2020 reviews by Izcovich and colleagues and July and colleagues reported 1.54× and 1.80× increase in mortality associated with dementia, respectively [13, 27], and three recent meta-analyses by Hariyanto and colleagues, Saragih and colleagues, and Damayanthi and colleagues concluded 2.62×, 2.96×, and 3.69× higher risk of mortality, respectively [9, 28, 29]. Contextually, our finding of 1.59× times higher mortality may seem low; however, the inclusion of multiple important confounders, e.g., entry oxygen saturation and pharmacological management, likely diminished the association but provided a “clearer” picture of the dementia-COVID link. In addition, our data suggests that the mortality is substantially increased even 1 year after discharge from the hospital setting, suggesting residual cognitive, pulmonary, or other ailments.

Additionally, the approach to dementia care in each "country" is a major confounder and could not be summarized by a singular number. Thus, our relatively moderate mortality risk may reflect a high standard of dementia care in Sweden and improvement in treatment and care of COVID-19. The mortality is substantially modified by increasing vaccination rates which was also likely reflected in the non-significant mortality increase in the third COVID-19 wave (the COVID-19 vaccination began at the end of December 2020 in nursing homes). Although the vaccination rates among dementia patients could be higher, Sweden in general has a high overall immunization percentage among the elderly [30], so this problem is likely non-differential.

While the higher risk of death in COVID-19-infected dementia patients may be intuitive, the underlying connections interacting between the two disorders are complex. First, patients with dementia are more prone to being infected and hospitalized with COVID-19 [7, 8, 15, 31, 32] and suffer more frequently from severe outcomes of COVID-19 [7, 9, 10]. These associations are likely explained by overall higher comorbidity burden including respiratory complications [11, 12, 33], inability to follow social restrictions [8, 15], increased caregiver burden [15], and possibly even higher infectious load in apolipoprotein E epsilon-4 variant carriers [15, 34]. Second, in older patients, the symptoms of the infection often do not conform to the usual clinical presentation, and atypical forms with altered mental status, tachypnea, and delirium may be dominant [8, 35]. In combination with lower social interaction, decreased frequency of health check-ups, lower cognitive ability to recognize the worsened state [15], and generally higher propensity towards pneumonia [12] may explain the more prevalent oxygen desaturation below 90% at admission in the dementia group and may have particularly contributed to the increased in-hospital mortality.

Furthermore, the clinical course of dementia is complicated by a variety of behavioral and psychological symptoms [36], and the burden of neuropsychiatric symptoms has increased during the pandemic [14, 37]. The increase is caused either indirectly through social isolation and lockdown measures [38, 39] or directly by COVID-19, which has been associated with the development and deterioration of neuropsychiatric sequelae [40]. Consequently, the presence of mental disorders may independently contribute to more severe outcomes in COVID-19 patients [41, 42], which would disproportionately impact patients with dementia.

Recent reports suggest the longitudinal functional and mental capacities in COVID-19 survivors may improve during the follow-up examinations [43, 44]; however, it is unclear whether such observations extend to dementia patients. Conversely, COVID-19 has been associated with exacerbations of cognitive and neurological symptoms [45, 46], which in the long term can be expected to result in an increase in future dementia cases, primarily through increasing the cardiovascular and cerebrovascular burden [47].

Therefore, the early identification of deviations from the normal clinical course and stricter observation of dementia patients are reasonable steps until the COVID-19 pandemic is completely under control.

In addition, a specific concern should be given to the finding of higher mortality among psychotropic drug users, particularly antipsychotics. Importantly, there are reports of increased antipsychotic drug prescription rates among dementia patients during the pandemic [16, 21] as well as associations between antipsychotics and higher probability of COVID-19 infection [32], though some reports dispute such claims [18]. In our cohort, the use of antipsychotic medication at admission to hospital was associated with 70% higher overall mortality, with the highest increase observed among in-hospital patients (3.58× higher hazard, with a 5% decrease in risk with each survived day), and the risk decreasing in the post-discharge periods (1.39× aggregate increase). The mortality increase may reflect the necessity of psychotropic medication use in overall more severe and atypically presenting COVID-19 (e.g., delirium, altered consciousness, and psychotic states) [8, 35, 48, 49], or higher psychiatric burden among patients with severe COVID-19, or necessary pharmacological management of BPSD. Importantly, the association survived adjustment for dementia, comorbidities, and oxygen saturation. Other studies concur with the direction of the association; however, the risk estimates vary between 1.26× and 11.1× the risk of death depending on the population, with large heterogeneity between the studied mortality periods [21, 22, 32, 44]. Unfortunately, we had no information on the pre-admission or post-discharge usage of antipsychotic drugs, which would allow us to confirm the putative increase in infection rates or study time-dependent effects.

The mortality risk in antipsychotics depends on the agents prescribed and dosage [20, 50, 51], which could have further confounded the association. We hypothesize that patients with dementia may be better adjusted to psychotropic medication due to BPSD, which may be reflected in the significantly higher psychotropic drug prescription among dementia patients. The results suggest some difference in risk estimates between dementia and dementia-free (38% in absolute difference, favoring dementia); however, the interaction was not significant. Sweden is a country with high quality of dementia care, and the care standards and the Swedish Dementia Registry (largest dementia registry in the world) [52] discourage the use of antipsychotics in dementia patients. Therefore, a comprehensive evaluation of psychotropic medication including dose-response is necessary before a valid guidance can be provided for individual drugs.

Further findings in our study confirm male sex as an independent predictor for higher mortality among COVID-19 patients with dementia, and the sex-stratified adjusted analyses suggested that the dementia-associated mortality increase has been observed primarily in male patients. Male sex is an established risk factor for higher COVID-19 mortality, with several putative factors suggested for the elevated risk (protective inflammatory effect of estrogens, higher comorbidity burden among men, behavioral/lifestyle differences) [53]. Importantly, the strength of the association in our study (55% increase) concurs with the findings of other authors [5, 13, 53] and suggests the male patients with dementia admitted for COVID-19 are more likely to need intensive treatment thus allowing for appropriate care allocation.

In conclusion, the study brings novel information on the short- and long-term mortality among the dementia population suffering from COVID-19. However, we would like to highlight the major role COVID-19 plays in the elderly population, and post hoc analyses of the pandemic’s impact on multimorbid and frail populations will be crucial [54].

Overall, our study provides robust evidence that dementia is a strong and independent risk factor for both short- and long-term mortality after COVID-19 infection, and the use of psychotropic medication, particularly antipsychotics, compounds the risk of dying. As the cognitive reserve is exhausted, even a mild form of COVID-19 will likely lead to a non-proportional decrease in cognitive functioning; therefore, dementia patients may benefit from heightened clinical vigilance and an individualized approach. Such efforts should be concurrent with the en-masse vaccination programs, particularly focusing on booster applications and repurposing the vaccines towards new variants of concern, where the immunization efficacy was not primarily studied [2, 55–58]. The improvement of social engagement, proper cardiovascular and cerebrovascular prevention, and cognitive training in the post-pandemic era will play a crucial role in the prevention of long-term damage in the already fragile population of dementia patients.

### Strengths and limitations

The main strengths of the study comprise the inclusion of a large unrestricted sample of geriatric patients with COVID-19 and the long follow-up after the hospitalization. The COVID-19 diagnosis was based on RT-PCR analysis of a nasopharyngeal swab specimen, generally considered the standard approach in COVID-19 diagnosis [59]. Moreover, the diagnosis of dementia was bi-specified by either the presence of ICD-10 codes or the use of anti-dementia medication, to increase the sample size. Another major strength is the division by periods of COVID-19 infection, where all three major infection waves were analyzed. Finally, the inclusion of comorbidity, medication, and clinical data allowed for more extensive adjustment and possible generalization to a larger population.

On the other hand, a careful interpretation of the medication findings is in order, as we had no information on pre-hospital medication usage, nor the indication data for the prescription. However, it is not clear how such misclassification would affect patients with dementia to a larger extent (specifically in the treatment with antipsychotics). In addition, the Swedish setting might not be representative of the antipsychotic medication usage elsewhere, as the two main clinical registries (Register for Behavioural and Psychological symptoms of dementia and Swedish Dementia Registry) advocate towards lower use of antipsychotics in dementia care. Conversely, this stresses the need for further reports from other European countries to enable comparisons.

Furthermore, we had no data on cognitive or functional performance measures in the patients; thus, no stratification or adjustment on dementia severity could have been made.

The inclusion of data only from the geriatric wards may limit the generalizability of the results to an older and more frail population; however, this should not alter the primary results that extend to the dementia population—a typical disorder of the advanced age. However, further post-discharge data are needed to comprehensively assess cognitive functioning in patients with dementia who overcame the COVID-19 infection.

Finally, as in all observational analyses, we acknowledge the possibility of residual and unknown confounding including the information on vaccination.

## Supplementary Information

**Additional file 1: Supplementary Figure 1.** Study sample selection.

**Additional file 2: Supplementary Table 1.** Baseline differences among the survivors and deceased during the whole follow-up. CCI, Charlson Comorbidity Index; RAAS, renin-angiotensin-aldosterone system; Age at admission is described as mean (SD); Other variables are described as n (%); SpO2 refers to the first oxygen saturation recorded during hospitalization; Attained comorbidities were recorded at admission and medication use was recorded at admission or at admission + 1 day; COVID-19 waves were divided based on dates - August 31^st^, 2020 (first and second wave) and February 28^th^, 2021 (second and third wave). Comparisons for scale variables across the dementia strata were performed using the independent-samples t-test (age at admission), and Mann-Whitney U-test (hospitalization duration). Chi-square test was used in all other descriptive analyses.

**Additional file 3: Supplementary Table 2.** Mortality rates and mortality rate ratios in COVID-19 patients with and without dementia. Mortality rate reflects the total deaths that occurred during the follow-up – during the first hospitalization and after discharge; COVID-19 waves were divided based on dates - August 31^st^, 2020 (first and second wave) and February 28^th^, 2021 (second and third wave).

## Data Availability

No data are available. The entities responsible for the original data and the Swedish law do not allow for sharing of the datasets containing the patient information.

## Abbreviations

ATC: Anatomical Therapeutic Chemical
CCI: Charlson Comorbidity Index
CI: Confidence interval
COVID-19: Coronavirus disease 2019
HR: Hazard ratio
ICD: International Classification of Disorders
RT-PCR: Reverse transcriptase-polymerase chain reaction
