# Supplementary figures and images for: Dementia and psychotropic medications are associated with significantly higher mortality in geriatric patients hospitalized with COVID-19: data from the StockholmGeroCovid project
Source: Alzheimers Res Ther. 2023 Jan 6;15:5. doi: 10.1186/s13195-022-01154-w (PMC9817345; doi:10.1186/s13195-022-01154-w)

Supplementary figure 1. Study sample selection


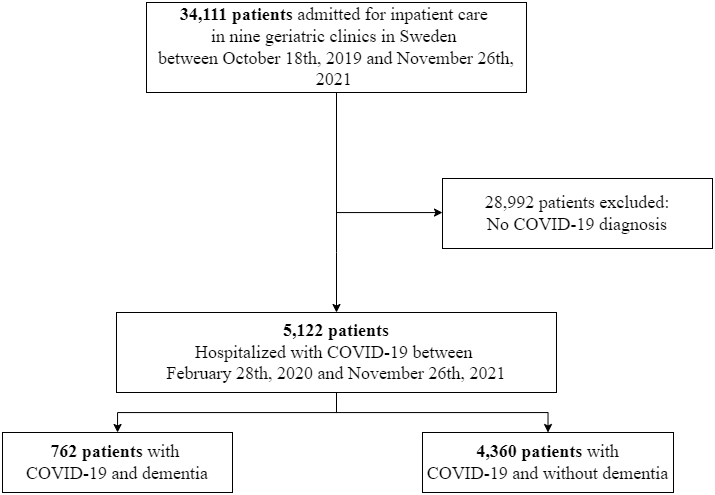

Supplement: Supplementary file 1 — Additional file 1: Supplementary Figure 1. Study sample selection. [file 13195_2022_1154_MOESM1_ESM.docx]
