# Supplementary material for: Dementia and psychotropic medications are associated with significantly higher mortality in geriatric patients hospitalized with COVID-19: data from the StockholmGeroCovid project
Source: Alzheimers Res Ther. 2023 Jan 6;15:5. doi: 10.1186/s13195-022-01154-w (PMC9817345; doi:10.1186/s13195-022-01154-w)
Supplement: Supplementary file 2 — Additional file 2: Supplementary Table 1. Baseline differences among the survivors and deceased during the whole follow-up. CCI, Charlson Comorbidity Index; RAAS, renin-angiotensin-aldosterone system; Age at admission is described as mean (SD); Other variables are described as n (%); SpO2 refers to the first oxygen saturation recorded during hospitalization; Attained comorbidities were recorded at admission and medication use was recorded at admission or at admission + 1 day; COVID-19 waves were divided based on dates - August 31st, 2020 (first and second wave) and February 28th, 2021 (second and third wave). Comparisons for scale variables across the dementia strata were performed using the independent-samples t-test (age at admission), and Mann-Whitney U-test (hospitalization duration). Chi-square test was used in all other descriptive analyses. [file 13195_2022_1154_MOESM2_ESM.docx]

Supplementary table 1. Baseline differences among the survivors and deceased during the whole follow-up

|  | | | Covid-19 patients  (First admission with COVID-19) | | |
| --- | --- | --- | --- | --- | --- |
|  | | | Deceased  (1,527) | Survivors  (3,595) | p |
| Age at admission, years | | | 85.7 (7.9) | 81.1 (8.3) | <0.001 |
| Age group | <70 | | 50 (3.3%) | 309 (8.6%) | <0.001 |
|  | 70-79 | | 283 (18.5%) | 1,238 (34.4%) |  |
|  | 80-89 | | 667 (43.7%) | 1,424 (39.6%) |  |
|  | >89 | | 527 (34.5%) | 624 (17.4%) |  |
| Male sex | | | 783 (51.3%) | 1,642 (45.7%) | <0.001 |
| CCI group | 0-1 points | | 772 (50.6%) | 2,376 (66.1%) | <0.001 |
|  | 2-3 points | | 499 (32.7%) | 959 (26.7%) |  |
|  | >3 points | | 256 (16.8%) | 260 (7.2%) |  |
| SpO_2_ | ≥90% | | 1,339 (89.2%) | 3,399 (95.6%) | <0.001 |
|  | <90% | | 162 (10.8%) | 155 (4.4%) |  |
| Dementia | | | 337 (22.1%) | 425 (11.8%) | <0.001 |
| Beta blockers | | | 854 (55.9%) | 1,740 (48.4%) | <0.001 |
| Ca2+ channel blocker | | | 407 (26.7%) | 1,139 (31.7%) | <0.001 |
| RAAS inhibitors | | | 708 (46.4%) | 1,831 (50.9%) | 0.003 |
| Statins | | | 532 (34.8%) | 1,615 (44.9%) | <0.001 |
| Antithrombotics | | | 1,474 (96.5%) | 3,465 (96.4%) | 0.80 |
| Antipsychotics | | | 256 (16.8%) | 306 (8.5%) | <0.001 |
| Antidepressants | | | 436 (28.6%) | 895 (24.9%) | 0.006 |
| Anxiolytics | | | 500 (32.7%) | 904 (25.1%) | <0.001 |
| Hypnotics/sedatives | | | 841 (55.1%) | 1,657 (46.1%) | <0.001 |
| COVID-19 wave | | First wave | 748 (49.0%) | 1,120 (31.2%) | <0.001 |
|  |  | Second wave | 595 (39.0%) | 1,501 (41.8%) |  |
|  |  | Third wave | 184 (12.0%) | 974 (27.1%) |  |
| Main cause of hospitalization | | COVID-19 | 1,283 (84.0%) | 3,103 (86.3%) | 0.098 |
|  |  | Cardiovascular | 51 (3.3%) | 83 (2.3%) |  |
|  |  | Respiratory | 47 (3.1%) | 84 (2.3%) |  |
|  |  | Neurological | 40 (2.6%) | 79 (2.2%) |  |
|  |  | External (trauma,poison) | 26 (1.7%) | 74 (2.1%) |  |
|  |  | Other | 80 (5.2%) | 172 (4.8%) |  |
| Hospitalization duration, days | | | 9 (9) | 8 (7) | <0.001 |

CCI, Charlson Comorbidity Index; RAAS, renin-angiotensin-aldosterone system; Age at admission is described as mean (SD); Other variables are described as n (%); SpO2 refers to the first oxygen saturation recorded during hospitalization; Attained comorbidities were recorded at admission and medication use was recorded at admission or at admission + 1 day; COVID-19 waves were divided based on dates - August 31^st^, 2020 (first and second wave) and February 28^th^, 2021 (second and third wave).

Comparisons for scale variables across the dementia strata were performed using the independent-samples t-test (age at admission), and Mann-Whitney U-test (hospitalization duration). Chi-square test was used in all other descriptive analyses.
