# Supplementary material for: Dementia and psychotropic medications are associated with significantly higher mortality in geriatric patients hospitalized with COVID-19: data from the StockholmGeroCovid project
Source: Alzheimers Res Ther. 2023 Jan 6;15:5. doi: 10.1186/s13195-022-01154-w (PMC9817345; doi:10.1186/s13195-022-01154-w)
Supplement: Supplementary file 3 — Additional file 3: Supplementary Table 2. Mortality rates and mortality rate ratios in COVID-19 patients with and without dementia. Mortality rate reflects the total deaths that occurred during the follow-up – during the first hospitalization and after discharge; COVID-19 waves were divided based on dates - August 31st, 2020 (first and second wave) and February 28th, 2021 (second and third wave). [file 13195_2022_1154_MOESM3_ESM.docx]

Supplementary table 2. Mortality rates and mortality rate ratios in COVID-19 patients with and without dementia.

| COVID-19  Dementia vs Dementia-free | | | | |
| --- | --- | --- | --- | --- |
|  | Deaths, n | Time at risk  (person-days) | Crude mortality rate (per 100 person-days) | Mortality rate ratio |
| Overall rates | | | | |
| Dementia | 337 | 193,285 | 0.17 | 1.88 |
| Dementia-free | 1,187 | 1,281,776 | 0.09 |  |
| First wave (28/2/2020 - 31/8/2020) | | | | |
| Dementia | 155 | 105,487 | 0.15 | 1.53 |
| Dementia-free | 593 | 617,796 | 0.10 |  |
| Second wave (1/9/2020 - 28/2/2021) | | | | |
| Dementia | 151 | 69,055 | 0.22 | 2.39 |
| Dementia-free | 442 | 483,107 | 0.09 |  |
| Third wave (from 1/3/2021) | | | | |
| Dementia | 31 | 18,743 | 0.17 | 1.96 |
| Dementia-free | 152 | 180,873 | 0.08 |  |

Mortality rate reflects the total deaths that occurred during the follow-up – during the first hospitalization and after discharge; COVID-19 waves were divided based on dates - August 31^st^, 2020 (first and second wave) and February 28^th^, 2021 (second and third wave).
